# Use of fast‐acting insulin aspart in insulin pump therapy in clinical practice

**DOI:** 10.1111/dom.13798

**Published:** 2019-06-19

**Authors:** Mark Evans, Antonio Ceriello, Thomas Danne, Christophe De Block, J. Hans DeVries, Marcus Lind, Chantal Mathieu, Kirsten Nørgaard, Eric Renard, Emma G. Wilmot

**Affiliations:** ^1^ Wellcome Trust/MRC Institute of Metabolic Science and Department of Medicine University of Cambridge Cambridge UK; ^2^ IRCCS MultiMedica Milan Italy; ^3^ Institut d'Investigacions Biomèdiques August Pi i Sunyer (IDIBAPS) Barcelona Spain; ^4^ Centro de Investigación Biomédica en Red de Diabetes y Enfermedades Metabólicas Asociadas (CIBERDEM) Madrid Spain; ^5^ Department of Cardiovascular and Metabolic Diseases IRCCS MultiMedica Sesto San Giovanni Italy; ^6^ Diabeteszentrum für Kinder und Jugendliche Kinderkrankenhaus auf der Bult Hannover Germany; ^7^ Department of Endocrinology‐Diabetology‐Metabolism Antwerp University Hospital Edegem Belgium; ^8^ Academic Medical Center University of Amsterdam Amsterdam The Netherlands; ^9^ Profil Institute of Metabolic Research Neuss Germany; ^10^ Department of Molecular and Clinical Medicine University of Gothenburg Gothenburg Sweden; ^11^ Department of Medicine NU ‐ Hospital Group Trollhättan/Uddevalla Sweden; ^12^ Clinical and Experimental Endocrinology University Hospital Leuven Leuven Belgium; ^13^ Steno Diabetes Center Copenhagen Gentofte Denmark; ^14^ Montpellier University Hospital, Department of Endocrinology, Diabetes, Nutrition and Institute of Functional Genomics University of Montpellier, CNRS, INSERM Montpellier France; ^15^ University Hospitals of Derby and Burton NHS Foundation Trust Derby UK

**Keywords:** CSII, insulin pump therapy

## Abstract

Fast‐acting insulin aspart (faster aspart) is a novel formulation of insulin aspart (IAsp) containing the additional excipients niacinamide and L‐arginine. The improved pharmacological profile and greater early glucose‐lowering action of faster aspart compared with IAsp suggests that faster aspart may be advantageous for people with diabetes using continuous subcutaneous insulin infusion (CSII). The recent onset 5 trial was the first to evaluate the efficacy and safety of an ultra‐fast‐acting insulin in CSII therapy in a large number of participants with type 1 diabetes (T1D). Non‐inferiority of faster aspart to IAsp in terms of change from baseline in HbA1c was confirmed, with an estimated treatment difference (ETD) of 0.09% (95% CI, 0.01; 0.17; *P* < 0.001 for non‐inferiority [0.4% margin]). Faster aspart was superior to IAsp in terms of change from baseline in 1‐hour post‐prandial glucose (PPG) increment after a meal test (ETD [95% CI], −0.91 mmol/L [−1.43; −0.39]; *P* = 0.001), with statistically significant improvements also at 30 minutes and 2 hours. The overall rate of severe or blood glucose‐confirmed hypoglycaemia was not statistically significantly different between treatments, with an estimated rate ratio of 1.00 (95% CI, 0.85; 1.16). A numerical imbalance in severe hypoglycaemic episodes between faster aspart and IAsp was seen in the treatment (21 vs 7) and the 4‐week run‐in periods (4 vs 0). Experience from clinical practice indicates that all pump settings should be reviewed when initiating faster aspart with CSII, and that the use of continuous glucose monitoring or flash glucose monitoring, along with a good understanding of meal content and bolus type, may also facilitate optimal use. This review summarizes the available clinical evidence for faster aspart administered via CSII and highlights practical considerations based on clinical experience that may help healthcare providers and individuals with T1D successfully initiate and adjust faster aspart with CSII.

## 1 INTRODUCTION

Continuous subcutaneous insulin infusion (CSII) using an insulin pump is an increasingly popular treatment option for children and adults with type 1 diabetes (T1D).^1^, ^2^, ^3^ In meta‐analyses of randomized controlled trials, CSII is associated with improved glycaemic control and lower risk of severe hypoglycaemia compared with multiple daily injection (MDI) therapy.^4^ CSII aims to mimic the physiological basal and prandial insulin profile, with basal infusion rates set to cover varying requirements during the night and between meals and user‐activated bolus doses at mealtimes.

Most insulin pumps offer a range of pre‐programmed bolus infusion types to provide coverage at mealtimes, including infusion of an entire bolus at once (standard bolus), infusion of small quantities over an extended period of time (delayed/extended bolus) or a combination of standard and delayed bolus (dual‐/multi‐wave).^5^, ^6^, ^7^ Insulin pumps also have integrated bolus calculators that enable insulin dose calculation based on carbohydrate counting, personalized carbohydrate:insulin ratios, duration of insulin action and insulin sensitivity factors, and they allow insulin doses to be adjusted by one tenth of a unit or less, compared with one unit or half a unit with pen injectors.

Despite developments in insulin pump technology, there are a number of challenges in optimizing glycaemic control with CSII. These include optimization of basal and bolus infusion rates, selection of bolus type, time of meal bolus programming, variability of insulin action and type of insulin used. Calculation of appropriate insulin doses requires users to perform frequent blood glucose testing (self‐measured blood glucose [SMBG]) at correct times or to use continuous glucose monitoring (CGM),^3^, ^8^, ^9^ and to make accurate estimations of meal composition and carbohydrate content.^10^, ^11^ Conventional insulin pumps involve an external infusion set to deliver insulin from the insulin reservoir in the pump housing into the subcutaneous tissue, while recently developed patch pumps deliver insulin via a very short internal infusion set.^12^ Pump failure, and infusion set malfunctions or occlusions, can cause unexplained hyperglycaemia, ketosis and diabetic ketoacidosis.^13^ The infusion site and the duration of infusion site usage can also impact the rate of insulin absorption and, consequently, the glucose‐lowering action.^14^

In normal physiology, insulin is secreted very rapidly from the β‐cell in response to, and even in anticipation of, a meal. Despite advances in insulin formulations, subcutaneously administered insulins have a delayed onset and a longer duration of action compared with endogenously secreted insulin. A recent study found a positive correlation between time‐to‐peak insulin action and HbA1c level in studies of closed‐loop insulin delivery and sensor‐augmented pump therapy, indicating the need for insulins with rapid and consistent absorption properties that are more able to reproduce physiological insulin responses.^15^ Current rapid‐acting insulin analogues (RAIAs) — insulin aspart (IAsp), insulin lispro and insulin glulisine — have faster absorption kinetics than regular human insulin;^16^ however, post‐prandial glucose (PPG) control with pump therapy remains limited by the pharmacokinetics of RAIAs.^17^

A new generation of ultra‐fast‐acting insulins, such as BioChaperone Lispro,^18^, ^19^ treprostinil lispro^20^ and fast‐acting insulin aspart (faster aspart), is under development. Faster aspart is the first of these to be approved for pump use in adults with T1D and type 2 diabetes (T2D) and it is now available in several countries. This review summarizes the available clinical data for faster aspart administered via CSII and highlights some practical considerations for its use in insulin pumps based on this evidence, as well as observations from clinical practice.

### 1.1 Fast‐acting insulin aspart (faster aspart)

Faster aspart is a novel formulation of IAsp containing the additional excipients niacinamide and L‐arginine.^21^ This novel formulation builds on the safety studies of conventional IAsp,^22^, ^23^ and both excipients are listed by the US Food and Drug Administration (FDA) as “generally recognized as safe” (GRAS).^24^ Niacinamide mediates faster initial absorption into the bloodstream by both increasing the initial abundance of IAsp monomers in the subcutaneous depot, and by mediating a transient, local vasodilatory effect;^25^ L‐arginine functions as a stabilizing agent.

In a pooled analysis of six clinical studies in adults with T1D, faster aspart administered via subcutaneous injection demonstrated an accelerated pharmacological profile compared with IAsp.^21^, ^26^ Faster aspart had an approximately 5‐minute earlier onset of appearance in the circulation, an approximately two‐fold higher early insulin exposure and an approximately 74% greater early glucose‐lowering effect within the first 30 minutes compared with IAsp.^26^ In addition, offset of exposure and glucose‐lowering effect occurred 12–14 minutes earlier with faster aspart than with IAsp. Similar pharmacological properties following subcutaneous injection have been observed in elderly adults and in a Japanese population,^27^, ^28^ as well as in children and adolescents with T1D.^29^

**Figure 1:**
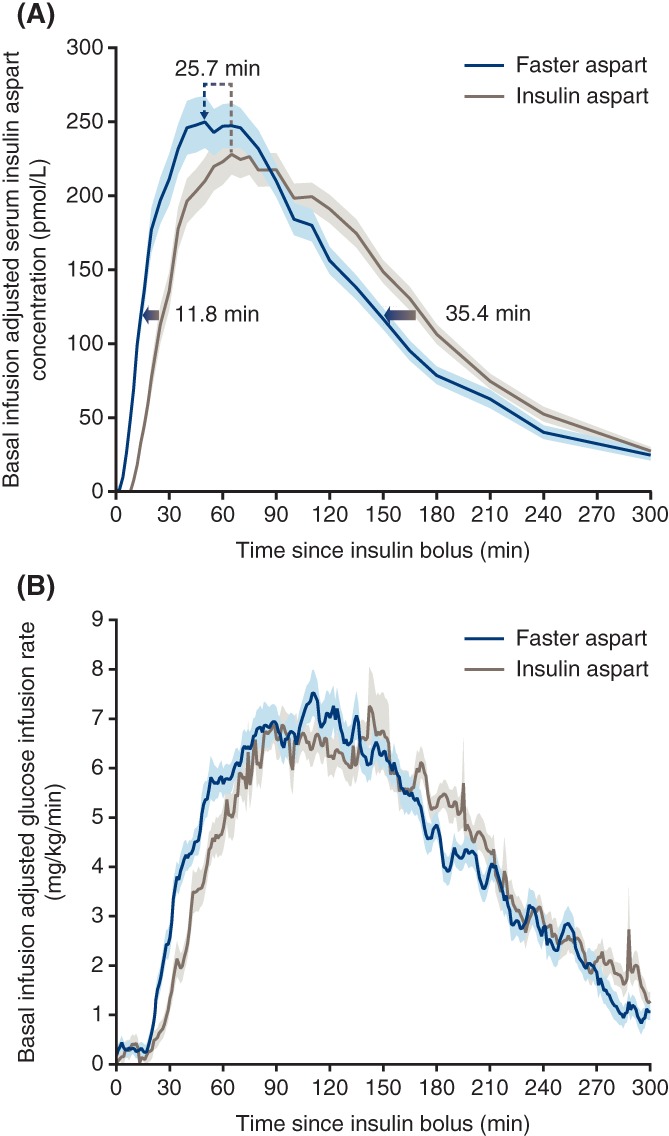
Key pharmacokinetic and pharmacodynamic properties of faster aspart administered via continuous subcutaneous insulin infusion. A, Mean serum insulin aspart concentration after bolus dose of 0.15 U/kg faster aspart or insulin aspart. Arrows indicate that the estimated onset and offset of exposure occurred earlier for faster aspart vs insulin aspart and show the left‐shift of the time of maximum insulin aspart concentration observed for faster aspart vs insulin aspart. B, Mean glucose‐lowering effect after bolus dose of 0.15 U/kg faster aspart or insulin aspart. Variability bands show the SEM. Abbreviations: Faster aspart, fast‐acting insulin aspart; SEM, standard error of the mean. Figure reproduced and adapted from Heise et al. *Diabetes Obes Metab*. 2017;19:208‐215,^30^ under the terms of Creative Commons Attribution‐NonCommercial‐License, © 2016

When delivered via CSII, the left‐shift in the pharmacological profile of faster aspart vs IAsp appears to be even greater compared with that seen after subcutaneous injection (Figure 1). In adults with T1D using CSII, faster aspart demonstrated an approximately three‐fold higher early insulin exposure and an approximately 100% greater glucose‐lowering effect within the first 30 minutes compared with IAsp.^30^ In addition, offset of exposure and offset of glucose‐lowering effect occurred 35 and 24 minutes earlier, respectively, with faster aspart than with IAsp. The reason for the differences between subcutaneous and CSII administration is not completely understood, and comparisons across trials should always be undertaken with caution; however, one hypothesis is that the continuous supply of niacinamide in a CSII setting further augments the rate of insulin monomer dissociation, thereby further increasing the early absorption rate of faster aspart compared with conventional IAsp. It is also possible that the smaller size of the CSII subcutaneous insulin depot, compared with a bolus injection, contributes to the accelerated kinetics of faster aspart versus IAsp.

## 2 CLINICAL EVIDENCE FOR FASTER ASPART

### 2.1 Multiple daily injection regimens

Several clinical trials comparing faster aspart and IAsp in MDI regimens demonstrate that the improved pharmacological properties of faster aspart translate into clinical benefits.^31^, ^32^, ^33^ The onset 1 and onset 8 trials in individuals with T1D reported non‐inferiority of MDI with mealtime faster aspart (administered 0–2 minutes before a meal) and post‐meal faster aspart (administered within 20 minutes after a meal) compared with IAsp in terms of HbA1c reduction 26 weeks after randomization, with a statistically significantly greater reduction with mealtime faster aspart in the onset 1 trial (onset 1: estimated treatment difference [ETD], −0.15% [95% CI, −0.23; −0.07], −1.62 mmol/mol [−2.50; −0.73]; onset 8: ETD [95% CI], −0.02% [−0.11; 0.07], −0.24 mmol/mol [−1.24; 0.76]). Mealtime faster aspart was also effective in reducing PPG excursions in both trials, and superiority to IAsp was confirmed (onset 1: 2‐hour PPG increment: ETD, −0.67 mmol/L [−1.29; −0.04], −12.01 mg/dL [−23.33; −0.70]; onset 8: 1‐hour PPG increment: ETD, −0.90 mmol/L [−1.36; −0.45], −16.24 mg/dL [−24.42; −8.05]). In both trials, the overall rate of severe or blood glucose (BG)‐confirmed hypoglycaemia (plasma equivalent glucose value <3.1 mmol/L [56 mg/dL]) was not statistically significantly different between mealtime or post‐meal faster aspart and IAsp, and the overall safety profiles were similar between treatments. A pooled post hoc analysis across both the onset 1 and onset 8 trials demonstrated a lower rate of nocturnal hypoglycaemia with mealtime faster aspart vs IAsp (estimated treatment ratio: 0.84 [95% CI, 0.72; 0.98]).^34^

### 2.2 Continuous subcutaneous insulin infusion setting

A small, exploratory, crossover trial demonstrated improvements in glycaemic control with faster aspart vs IAsp in adults with T1D using CSII,^35^ with an approximately 25% greater glucose‐lowering effect during the first 2 hours following a standardized meal test (ETD, −0.99 mmol/L [95% CI, −1.95; −0.03], −17.84 mg/dL [−35.21; −0.46]). This was supported by 2 weeks of CGM data, which indicated improvements in PPG control after all regular meals with faster aspart vs IAsp, with the largest difference at breakfast (1‐hour interstitial glucose [IG] increment, 1.12 vs 2.04 mmol/L [20.19 vs 36.69 mg/dL], respectively).

Insulin preparation formulation type can influence the risk of infusion set failure;^36^ however, results of the 6‐week onset 4 trial indicated a similar compatibility of faster aspart and IAsp with CSII.^37^ No microscopically‐confirmed infusion set occlusions were observed for faster aspart or IAsp and, after adjusting for an imbalance during the run‐in period, the rate of severe or BG‐confirmed hypoglycaemia was similar for both insulins. A higher number of premature infusion‐set changes was observed with faster aspart vs IAsp (21 changes reported by 11 participants vs four reported by two participants, respectively), with technical issues being the most commonly cited reason. As this was a relatively small trial of short duration, further studies may be needed to get a true feel for insulin pump compatibility.

The recent double‐blind, treat‐to‐target, randomized, 16‐week onset 5 trial evaluated the efficacy and safety of faster aspart administered via CSII in 472 adults with T1D.^38^ During the 4‐week run‐in period, participants received reinforcement of training in pump use, diabetes education and explanation of trial procedures; and mean HbA1c decreased from 7.79% and 7.80% in the faster aspart and IAsp treatment arms, respectively, to 7.49% in both arms. Participants continued using their pre‐trial insulin (3% insulin glulisine, 40% insulin lispro and 57% IAsp), and basal pump rates and bolus dose calculator settings were not adjusted unless necessary for safety reasons. At randomization, participants switched to double‐blinded treatment with faster aspart or IAsp on a unit‐for‐unit basis. Basal rates were adjusted to target a fasting and pre‐prandial SMBG between 4.0 and 6.0 mmol/L (71–108 mg/dL) (plasma‐equivalent glucose values) and to ensure that fasting plasma glucose was maintained within a stable range (within 2 mmol/L [35 mg/dL]), and mealtime insulin (administered 0–2 minutes before a meal) was titrated based on carbohydrate counting. Participants continued using their own insulin pump, and approximately 25% of participants in each treatment arm used their own real‐time CGM device. During the treatment period, HbA1c decreased further to 7.44% in the faster aspart arm and to 7.35% in the IAsp arm. As expected with a treat‐to‐target design, non‐inferiority between treatments was confirmed with regard to change in HbA1c; however, the ETD was statistically significant in favour of IAsp (Table 1).^38^ In contrast, PPG increments at 30 minutes, 1 and 2 hours after a standardized meal test were statistically significantly reduced with faster aspart compared with IAsp. This was corroborated by lower post‐prandial IG increments after 1 and 2 hours with faster aspart vs IAsp, measured during three approximately 2‐week periods of blinded CGM (Table 1).^38^

**Table 1:** Faster aspart in continuous subcutaneous insulin infusion: Key efficacy and safety endpoints from the onset 5 trial

Efficacy	Faster aspart	Insulin aspart	Estimated treatment difference (95% CI), *P‐*value^a^
HbA1c 16 weeks after randomization *(primary endpoint)*, %	7.44	7.35	0.09 (0.01; 0.17), *P* = 0.022 *(non‐inferiority confirmed,* *P <* 0.001^b^ *)*
Change from baseline 16 weeks after randomization			
30‐min PPG increment (meal test), mmol/L	−0.53	0.11	−0.66 [−1.00; −0.31], *P* < 0.001
1‐h PPG increment (meal test), mmol/L *(confirmatory secondary endpoint)*	−0.89	0.05	−0.91 [−1.43; −0.39], *P* = 0.001 *(superiority confirmed, P <* 0.001^b^ *)*
2‐h PPG increment (meal test), mmol/L	−0.82	0.09	−0.90 [−1.58; −0.22], *P* = 0.01
0–1‐h IG increment (CGM), mmol/L			
Breakfast	−0.13	0.14	−0.27 [−0.44; −0.11], *P* = 0.001
Lunch	−0.02	0.15	−0.20 [−0.35; −0.06], *P* = 0.004
Main evening meal	−0.16	0.04	−0.15 [−0.28; −0.01], *P* = 0.032
All meals	−0.10	0.11	−0.21 [−0.31; −0.11], *P* < 0.001
0–2‐h IG increment (CGM), mmol/L			
Breakfast	−0.28	0.16	−0.43 [−0.67; −0.18], *P* = 0.001
Lunch	−0.24	0.22	−0.44 [−0.65; −0.23], *P* < 0.001
Main evening meal	−0.29	−0.03	−0.23 [−0.43; −0.04], *P* = 0.018
All meals	−0.25	0.12	−0.38 [−0.52; −0.23], *P* < 0.001
**Safety**	**Faster aspart**	**Insulin aspart**	**Estimated treatment ratio (95% CI)**
Hypoglycaemic episodes, PYE			
Severe or BG‐confirmed			
Overall	45.07	45.29	1.00 (0.85; 1.16), NS
Within 1 h	1.26	0.71	1.78 (1.15; 2.75), *P* = 0.009
>1–2 h	5.36	5.05	1.05 (0.82; 1.35), NS
>2–3 h	6.78	7.76	0.86 (0.70; 1.06), NS
>3–4 h	5.95	6.03	0.98 (0.77; 1.24), NS
Severe			
Treatment period	0.29	0.10	2.78 (0.78; 9.94), NS
Run‐in	0.21	0.00	‐
Infusion‐site reactions, PYE			
All	0.61	0.45	‐
Possibly or probably related to trial product	0.29	0.18	‐
Infusion set changes, PYE			
All	132.67	130.57	‐
Non‐routine changes	6.97	6.68	‐

Abbreviations: BG‐confirmed, recorded plasma equivalent glucose value <3.1 mmol/L (56 mg/dL); CGM, continuous glucose monitoring; CI, confidence interval; faster aspart, fast‐acting insulin aspart; IG, interstitial glucose; NS, not significant; PPG, post‐prandial glucose; PYE, number of events per patient‐year of exposure.

a *P* values from a two‐sided test for treatment difference evaluated at the 5% level.

b *P* values from a one‐sided test for non‐inferiority and superiority evaluated at the 2.5% level.

The reasons for the discrepancy between the impact on HbA1c levels and PPG control are not fully clear. Participants did not change pump settings during the double‐blinded trial period unless deemed necessary by an investigator; thus, pump parameters were optimized for RAIAs rather than faster aspart use. Nocturnal and pre‐meal levels of IG were slightly higher in participants receiving faster aspart compared with those receiving IAsp. Elevated nocturnal IG in the faster aspart treatment arm may have been the result of a suboptimal bolus type (ie, dual‐/multi‐wave vs standard bolus) for the composition of the evening meal (eg, fat content), a lack of basal insulin compensation because of the shorter bolus insulin action, or suboptimal basal insulin rates during the night.

The rate of overall severe or BG‐confirmed hypoglycaemia was not different between treatments; however, consistent with its faster pharmacokinetic/pharmacodynamic (PK/PD) profile, the rate of the small proportion of episodes that occurred during the first hour after a meal was higher for faster aspart vs IAsp (Table 1). While the trial was not powered to assess differences in severe hypoglycaemia, the number of episodes was numerically higher for faster aspart vs IAsp. Eleven participants treated with faster aspart reported 21 episodes and five participants treated with IAsp reported seven episodes. This imbalance was also observed during the run‐in period, with four episodes reported by three participants who were later randomized to faster aspart. Unlike many clinical trials of CSII, it is important to note that individuals with hypoglycaemia unawareness or with preceding severe hypoglycaemia were not excluded from this trial, and there was no stratification for these parameters. A similar rate of infusion set changes (routine and non‐routine) was reported with both treatments, although a numerically higher number of infusion‐site reactions, a cited reason for non‐routine changes, was reported with faster aspart vs IAsp (Table 1).

### 2.3 Closed‐loop automated insulin delivery systems

Many hybrid and fully closed‐loop insulin delivery systems have been limited by the degree of aggressiveness with which RAIAs can be used to control PPG because of the risk of late hypoglycaemia. The use of a faster‐acting insulin in these systems is expected to be of great interest, and trials of faster aspart are in progress.^39^, ^40^, ^41^ Indeed, the observed elevated nocturnal IG reported with faster aspart in the onset 5 trial could potentially be minimized by the automated basal insulin delivery offered by closed‐loop systems. An initial study suggests that faster aspart provides a modestly greater glucose‐lowering effect compared with IAsp in a fully closed‐loop delivery system (ΔAUC_0–1h_ after breakfast, −3782 mmol/L*min; ΔAUC_0–5h_ after dinner, −1158 mmol/L*min).^42^ At present, closed‐loop glucose control algorithms are designed for use with RAIAs and the more rapid onset of faster aspart may require adaptations of these algorithms. Clinical trials will be required to provide an answer to this important question.

## 3 FASTER ASPART VIA CSII: PRACTICAL CONSIDERATIONS

The accelerated absorption kinetics of faster aspart suggest that it would provide clinical benefits with use of CSII. Despite improvements in PPG control, it is surprising that treatment with faster aspart did not improve HbA1c to a greater extent than treatment with IAsp in the onset 5 trial. The double‐blind design of the onset 5 trial prevented tailored adjustments according to the pharmacological profile of faster aspart, and conventional CSII practices may require optimization to realize fully the potential benefits of faster aspart. Faster aspart has been approved for use in insulin pumps for CSII by the European Medicines Agency and is available in several countries.^43^ However, practical guidance on the use of faster aspart with CSII is lacking. Herein, we highlight important considerations that may aid healthcare providers (HCPs) and individuals with diabetes in successfully initiating and adjusting faster aspart with CSII. A number of these considerations will probably also apply to other ultra‐fast‐acting insulins currently in development.

As in the onset 5 trial, a 1:1 unit dose conversion is recommended when switching to faster aspart. However, while pump settings may have been ideal for the previously used insulin, given the difference in pharmacology, a review of and guided change in all pump settings should be expected over the weeks and months following the switch. Differences in bolus delivery between different insulin pumps should also be taken into consideration, as these can affect the pharmacological characteristics of mealtime insulin^44^ and may also influence the “insulin on board” or active insulin estimation, that is, the residual glucose‐lowering activity from prior boluses, and therefore, the correction bolus dosing.

Because of the accelerated absorption kinetics of faster aspart, bolus dosing will need to be addressed to reduce the risk of early post‐prandial hypoglycaemia or late post‐prandial hyperglycaemia. Early post‐prandial hypoglycaemia is uncommon, but it may become an issue after unexpectedly delayed meals or meals with a high fat content, errors in carbohydrate counting, or in patients with gastroparesis. Data suggest that administration of a pre‐prandial bolus of ultra‐rapid‐acting insulin 15 minutes before a meal, compared with immediately before, can improve post‐prandial hyperglycaemia.^45^ While this was not examined in the onset 5 trial, clinical experience suggests that pre‐meal bolus dosing can be beneficial for pump users with faster aspart, especially when consuming food with a high glycaemic index. Adjustments to the basal insulin dose, potentially using a basal rate test, will also need to be taken into consideration for optimal use of faster aspart,^46^ although HCPs should be aware that some pump users will not be accustomed to changing basal rate parameters without support from their treatment team.

Pump users should monitor BG adequately and may need to increase the frequency of SMBG testing to enable optimization. The use of CGM or flash glucose monitoring (FGM) could enable optimization of dosing for each individual user when switching to faster aspart. If long‐term use of CGM or FGM is not possible, short‐term use over 8–12 weeks would probably be helpful. Monitoring the “insulin on board”/active insulin function of the pump could help pump users understand and tailor their dosing needs.

**Figure 2:**
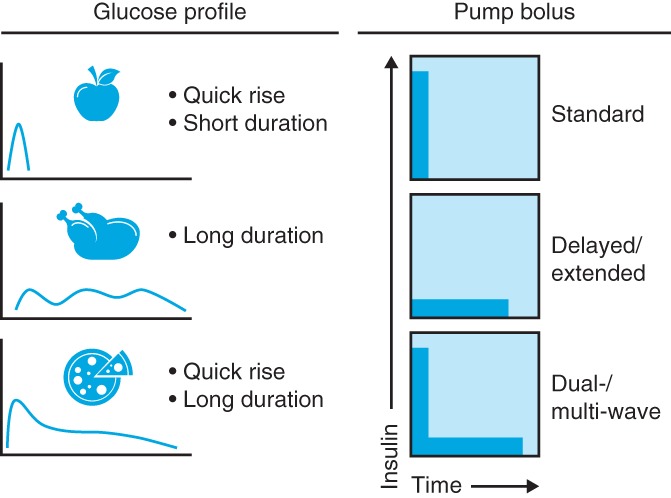
Considerations for bolus type with mealtime insulin. Consideration should be given to matching the type of bolus insulin administered via a pump with the expected glucose profile of a meal

A good understanding of meal content and the glycaemic index is probably important for pump users to fully benefit from the effect of faster aspart. Although the use of faster aspart in the context of high‐ or low‐glycaemic index meals has not been addressed in clinical trials, there may be a need for different bolus types, such as a delayed/extended bolus with larger meals or a dual‐ or multi‐wave bolus for high‐fat and high‐protein meals (Figure 2).^47^, ^48^, ^49^ As a starting point for high‐fat and high‐protein meals, 30% of the total insulin dose can be administered immediately and 70% administered with a delay over the 2–4 hours following the meal. It should also be noted that more insulin than that calculated by carbohydrate counting alone may be needed.^49^, ^50^

The occurrence of a burning sensation around the infusion site has been reported in some individuals using faster aspart in clinical practice. Some users also report the necessity of changing their infusion set more frequently after switching to faster aspart to avoid hyperglycaemia, and others have found that correction doses are not as effective as expected. There are likely to be other, currently unknown, factors involved in determining the success of treatment with faster aspart using CSII, and HCPs may find that improvements in glycaemic control are seen in some users, but not necessarily all.

## 4 SUMMARY

Use of faster aspart in insulin pump therapy provides potential benefits for glycaemic control. The improved PK/PD profile of faster aspart compared with that of IAsp suggests that faster aspart may be advantageous for individuals with diabetes using CSII. While the large, double‐blind onset 5 trial demonstrated that faster aspart is effective in glycaemic control, superiority of faster aspart over IAsp in terms of HbA1c reduction was not confirmed, although meal test and CGM results suggest that faster aspart is especially beneficial for PPG control. Experience from clinical practice indicates that initiating faster aspart with CSII should not be viewed as a simple switch of insulin. All pump settings will need to be reviewed and tailored to the individual patient. The use of CGM or FGM, along with a good understanding of meal content and bolus type, may also facilitate optimal use of faster aspart with CSII. There is currently limited evidence concerning the clinical use of faster aspart with CSII, and further studies are required to maximize its potential benefits in pump therapy.

## CONFLICT OF INTEREST

M. E. has served on advisory boards for Novo Nordisk, Abbott Diabetes Care, Roche, Medtronic, CellNovo and Dexcom; has received speaker's fees from Novo Nordisk, Abbott Diabetes Care, Eli Lilly, MSD and AstraZeneca; has received travel support from Novo Nordisk; and has participated in research collaborations/trial list with MedImmune, Boehringer Ingelheim, Novo Nordisk and Sanofi. A. C. has received research grants from AstraZeneca, Novartis and Mitsubishi and personal fees from AstraZeneca, Boehringer Ingelheim, DOC Generici, Eli Lilly, Janssen, Novo Nordisk, OM Pharma, Novartis, Sanofi and Takeda.

T. D. has received research support from or has consulted for Abbott, AstraZeneca, Bayer, Boehringer, DexCom, Insulet Corp., Eli Lilly, Medtronic, Novo Nordisk, Roche and Sanofi; and is a shareholder in DreaMed.

C. D. B. has received personal fees from Abbott, AstraZeneca, A. Menarini Diagnostics, Johnson & Johnson, Lilly, Medtronic, MSD, Novartis, Novo Nordisk, Roche Diagnostics and Sanofi.

J. H. D. V. serves on advisory panels for Insulet, Novo Nordisk, Roche, Sanofi and Zealand Research; has received research support from Dexcom, Medtronic, Novo Nordisk and Senseonics; and has received speaker's fees from Novo Nordisk, Roche and Senseonics.

M. L. has received research grants from AstraZeneca, DexCom and Novo Nordisk; and has received honoraria from or consulted for AstraZeneca, DexCom, Eli Lilly, MSD and Novo Nordisk.

C. M. has served on advisory panels for Novo Nordisk, Sanofi‐Aventis, Merck Sharp & Dohme Ltd., Eli Lilly and Company, Novartis, AstraZeneca LP, Jansen Pharmaceuticals, Hanmi Pharmaceuticals, Intrexon, Boehringer Ingelheim; has received research support from Novo Nordisk, Sanofi‐Aventis, Merck Sharp & Dohme Ltd., Boehringer Ingelheim; and has participated in speakers' bureaus for Novo Nordisk, Sanofi‐Aventis, Merck Sharp & Dohme, Eli Lilly and Company, Novartis and AstraZeneca.

K. N. serves as an adviser at Medtronic, Abbott and Novo Nordisk; owns shares in Novo Nordisk; has received research grants from Novo Nordisk, Zealand Pharma and Roche; and has received speaker's fees from Medtronic, Roche, Rubin Medical, Sanofi, Zealand Pharma, Novo Nordisk and Bayer.

E. R. serves as a consultant/advisor for Abbott, Air Liquide, Cellnovo, Dexcom, Eli Lilly, Insulet, Johnson & Johnson (Animas, LifeScan), Medtronic, Novo Nordisk, Roche Diagnostics and Sanofi‐Aventis; and has received research grants/material support from Abbott, Dexcom, Insulet, Roche Diagnostics and Tandem Diabetes Care.

E. G. W. has received personal fees from Abbott Diabetes Care, Dexcom, Eli Lilly, Medtronic, Novo Nordisk and Sanofi‐Aventis.

## AUTHOR CONTRIBUTIONS

All authors discussed the concept of the review, worked on the outline, commented in detail on the first iteration, made critical revisions on later drafts and approved the final draft for submission.
